# Towards the elimination of mother to child transmission of syphilis 2015–2020: practice and progress in Zhejiang province, eastern China

**DOI:** 10.1186/s12884-022-05258-x

**Published:** 2023-02-07

**Authors:** Hong Wang, Xia Ying, Dan Lin, Muhuza Marie Parfaite Uwimana, Xiaohui Zhang

**Affiliations:** 1 Department of Obstetrics, Women’s Hospital, School of Medicine, Zhejiang University, Hangzhou, Zhejiang China; 2 Department of Women’s Health, Women’s Hospital, School of Medicine, Zhejiang University, Hangzhou, Zhejiang China; 3 School of Medicine, Zhejiang University, Hangzhou, Zhejiang China

**Keywords:** Elimination of mother-to-child transmission (EMTCT), Adverse pregnancy outcomes (APOs), Screening, Treatment, Pregnant women

## Abstract

**Objectives:**

To estimate the progress towards elimination of mother-to-child transmission (EMTCT) of syphilis in Zhejiang province.

**Methods:**

Data were obtained from Zhejiang provincial EMTCT network. Childbearing women infected with syphilis during 2015–2020 were recruited. Joinpoint mode was used to analyze changing trends in syphilis screening, treatment and adverse pregnancy outcomes (APOs). Changing trends were presented as the annual percentage of change (APC). Multivariate logistic regression mode was used to analyzerisk factors of APOs.

**Results:**

Of 3,658,266 participants, an average maternal syphilis incidence was 0.38%. From 2015 to 2020, the coverage of syphilis screening in pregnancy (96.31% to 99.24%; P < 0.001) and coverage of antenatal health care (ANC) within 13 gestational weeks (55.27% to 77.82%; P = 0.002) were increased.The coverage of maternal syphilis treatment(88.30% to 98.25%; P = 0.001) and adequate treatment (66.92% to 83.37%; P = 0.001) were also increased. Over the years, the APC was -19.30% (95%CI:-24.33 ~ -13.92, P = 0.001) in perinatal death,-26.55% in congenital syphilis(95%CI:-38.75 ~ -11.92, P = 0.009), and -14.67% in other neonatal complications (95%CI:-23.96 ~ -4.24, P = 0.019).In 2020, 11.58% of women had APOs. The rate of syphilis infection during pregnancy increased among women aged (< 20 years) or (≥ 35 years), multiparous, and with pregnancy complications (all P < 0.05).APOs risk increased in women with higher maternal RPR/TRUST titers while it decreased in women who had (adequate) therapy, early ANC, and aged in 21–34 years (all *P* < 0.001).

**Conclusions:**

Despite steady progress towards the goal of EMTCT in implementing universal screening and treatment, syphilis continuously affects a large number of pregnant women. Increasing vulnerable women, small proportions of inadequately treated and delay in early ANC should be noticed.

## Introduction

The estimated worldwide prevalence of maternal syphilis in 2016 was 0.69% (95% confidence interval: 0.57%–0.81%) [1]. It has been estimated that women with syphilis account for 20%–60% of adverse pregnancy outcomes (APOs), and risk is higher for those who are unscreened, untreated, or inadequately treated [1–4]. Maternal syphilis and associated APOs are preventable with adequate penicillin treatment [5]. In 2007, WHO advocated eliminating mother-to-child transmission of syphilis (EMTCT) [6]. With the improvement of syphilis screening and treatment in pregnant women, several countries such as Thailand and the Republic of Cuba have already achieved the EMTCT goals [7, 8].

In 2010 the Ministry of Health (MOH) of China made an announcement of a national Plan for Syphilis Control and Prevention (2010–2020) under the call of global EMTCT [9]. In 2011, Guidelines for Prevention of Mother-to-Child Transmissions of HIV, Syphilis, and Hepatitis B virus (HBV) were integrated and implemented, which set an overall target of achieving 95% syphilis-screening coverage; 90% syphilis-screening coverage during pregnancy; 90% intervention coverage for infected pregnant women and their babies, and an incidence of congenital syphilis under 15 per 100,000 live births by 2020 [9]. In 2017, the Chinese government launched a pilot project of EMTCT, and Zhejiang was appointed as one of the three pilot provinces.

Zhejiang province is located on the southeast coast of China, has a high gross domestic product (GDP) and large migrant population [10–12]. Additionally, the burden of maternal syphilis is critical in Zhejiang province, as 2013- 2014 data shows that the average incidence was 0.3% [13]. This study aimed to explore changing trends in maternal syphilis screening, treatment, APOs along with related risk factors since 2015.This study will update our previous research of 2013–2014 and estimate the progress towards achieving the goals of EMTCT of syphilis [13]. Our findings would contribute to significant further guidance on maternal and newborn health Sustainable Development Goals.

## Methods

### Study design and data source

The retrospective study obtained data from the Zhejiang provincial EMTCT information network, which is a mandatory case reporting system covering all health care institutions and delivery hospitals. In this study, we recruited pregnant women with syphilis who gave live births or stillbirths from 2015 to2020. Information on maternal socio-demographic characteristics, ANC service, syphilis related screening and treatment, pregnancy outcomes, and birth information were collected through a web-based information system. Quality control is performed by experts at the local level and provincial level, involving ANC service, laboratory quality, data collection, and associated therapy. We defined stillbirth as fetal death at or more than 28 gestational weeks, prior to delivery and before onset of labor., or at least 1000 g weight. Study size was calculated by simple random sampling formula as follow.

$$n=\frac{{Z}_{\alpha /2}^{2}p\left(1-p\right)N}{{\delta }^{2}\left(N-1\right)+{Z}_{\alpha /2}^{2}p\left(1-p\right)N}$$

P was defined as the overall rate of APOs (20%). $\delta$ = 0.05(forgives errors), $\alpha$= 0.05(examination standard).

The minimum study size of maternal syphilis was 246. Therefore, the total number of maternal syphilis at provincial level is powerful enough to identify the APOs in the study.

### Introduction of EMTCT in Zhejiang province

#### Screening

In Zhejiang province, pregnant women are required to receive first antenatal care ANC within 13 gestational weeks. An integrated HIV, syphilis, and HBV free screening program is provided to all pregnant women at first ANC and time of delivery. Syphilis rapid plasma regain (RPR), or toluidine red unheated serum test (TRUST), and Treponema Pallidum Hemagglutination Assay (TPHA) test or Treponema pallidum particle agglutination (TPPA) are used for detection of maternal syphilis. If necessary, laboratory confirmation of *Treponema pallidum* in clinical specimens by dark-field microscopy or reactive treponemal IgM antibody test ares performed.

#### Treatment

Benzathine penicillin (2.4 million units) by weekly injection for 3 weeks or procaine penicillin (0.8 million units) by daily injection for 15 days per treatment course is offered as the first option for pregnant women with syphilis. Either ceftriaxone (1 g per day) for 10 days or erythromycin (500 mg/4 times per day) for 15 days is suggested for penicillin allergy. Women infected with syphilis are followed up throughout pregnancy and postpartum by in woman’s and child’s healthcare centers or hospitals at regional level. Women with reoccurrence or secondary infection repeat treatment.

#### Neonatal syphilis( screening, diagnosis and treatment)

Newborns meeting any of the following criteria are diagnosed with congenital syphilis (CS): (1) Positive treponemal test and a value of fourfold higher titer of nontreponemal test than that of his/her mother’s before delivery; (2) laboratory confirmation of *T. pallidum* in clinical specimens by dark-field microscopy; (3) reactive treponemal antibody test of IgM. These criteria are consistent with those mentioned in other literatures from China [10, 14]. Infants diagnosed with CS and infants born to inadequately treated women are also offered free penicillin treatment.

Since the inception of the EMTCT program in 2017, Zhejiang province has taken a series of measures to enhance the program towards WHO EMTCT goals, ensuring treatment of all pregnant women with syphilis, advocating social support, upholding human rights, training for healthcare providers and reviewing MTCT failed cases.

### Variable definition

#### Coverage

The overall coverage of syphilis screening in pregnant women meant the number of syphilis screening during pregnancy or at delivery per 100 pregnant women. Moreover, we also describedthe coverage of syphilis screening during pregnancy per 100 women by excluding women tested at delivery.

#### APOs

The overall incidence of APOs was defined as births with any of the following: low birth weight (LBW, birth weight under 2,500 g), preterm birth (born at or more than 28 gestational weeks but before 37 gestational weeks), perinatal death (fetal death ≥ 28 gestational weeks or new infant death within 7 days after birth), CS and other neonatal complications (birth defects, neonatal pneumonia, asphyxia neonatorum).

#### Treatment

Overall treatment indicated women being treated during pregnancy or at delivery, including penicillin, ceftriaxone, or erythromycin. Adequate maternal syphilis treatment was defined as complete penicillin treatment (2-course treatment, the interval between the 2 courses ≥ 2 weeks, provided at least 1 month prior to delivery). To evaluate maternal syphilis treatment adequacy or re-infection before delivery or third trimester, RPR/TRUST results were divided into four groups according to maternal titres (titres ≥ 1:32, titres = 1:16, titres = 1:8 and titres ≤ 1:4).Pregnant women with higher titers were treated again.

Statistics Chi-square test was used to analyze variables’distributions. Joinpoint Regression mode was used for changing trends analysis over the years to calculate annual percentage change and 95% confidence intervals (APC, 95% CI).The risk of APOs was calculated using logistic regression mode with adjusted odds ratio presented together with 95% confidence intervals (OR and 95% CI). All P value under 0.05 were regarded as statistically significant. SPSS 20.0(IBM SPSS Statistics for Windows, Version 27.0.Armonk, NY; IBM Corp), Stata version 13 (Stata Corp, College Station, TX, United States)and Joinpoint software (Version 4.7.0.0—February 2019; Statistical Methodology and Applications Branch, Surveillance Research Program, National Cancer Institute) were used for statistical analysis.

### Ethics approval and data availability

The study was performed in accordance with the Declaration of Helsinki and approved by Women’s Hospital School of Medicine Zhejiang University ethics committee (No.20180180). The need for informed consent was waived by the ethics committee Review Board of Women’s Hospital School of Medicine Zhejiang University ethics committee, because of the retrospective nature of the study. Data are available upon reasonable request.

## Results

### Changes in syphilis screening and incidence in pregnant women

During 2015–2020, the total number of pregnant women included in the analysis was 3,658,266. Of them, 3,655,371 had syphilis screening during pregnancy, and the rest were screened at time of delivery. The overall coverage of syphilis screening in pregnant women remained high throughout the study period(P = 0.870). Moreover, the coverage of syphilis screening during pregnancy increased significantly from 96.31% in 2015 to 99.24% in 2020 (APC = 0.60, 95%CI:0.48 ~ 0.71, P < 0.001), and the coverage of ANC in the first trimester increased significantly from 55.27% to 77.82% (APC = 6.71, 95%CI:4.17 ~ 9.31, *P* = 0.002). Totally, 13,829 women were diagnosed with syphilis, giving an average incidence of 0.38%. The maternal syphilis incidences were stable over years (APC = 2.08, 95%CI:-2.79 ~ 7.19,P = 0.307), with 0.44% in 2020 (Table 1).

**Table 1:** Changes in coverage of syphilis screening, first antenatal health care visit and incidences of maternal syphilis during 2015–2020

Year	Number of pregnant women (a)	Total number of pregnant women with syphilis screening (b)	Overall coverage of syphilis screening in pregnant women (b/a, %)	Number of syphilis screening in women during pregnancy (c)	Coverage of maternal syphilis screening during pregnancy (c/a, %)	Number of women with syphilis (d)	Incidence of maternal syphilis (d/b, %)	Number of women with syphilis who had ANC in first trimester (e)	Coverage of Women with syphilis with ANC in first trimester (e/d,%)
2015	574,030	573,185	99.85	552,867	96.31	2,231	0.39	1,233	55.27
2016	724,485	723,400	99.85	703,646	97.12	2,520	0.35	1,551	61.55
2017	704,024	704,007	100.00	688,063	97.73	2,647	0.38	1,809	68.34
2018	602,107	601,257	99.86	590,344	98.05	2,240	0.37	1,618	72.23
2019	582,227	582,173	99.99	575,995	98.93	2,135	0.37	1,584	74.19
2020	471,393	471,349	99.99	467,819	99.24	2,056	0.44	1,600	77.82
Total	3,658,266	3,655,371	99.99	3,578,734	97.83	13,829	0.38	9,395	67.94

### Changes in the characteristics of women with syphilis

During 2015–2020, the characteristics distribution of women with syphilis significantly differed in maternal age (P < 0.001), maternal education(P < 0.001), multipara(P < 0.001), marital status(P < 0.001), local residents or not(P < 0.001), with or without pregnancy complications (P = 0.031), and maternal RPR/TRUST serological titers(P < 0.001). Overall, most women were unemployed (51.04%), with secondary education (75.95%), married (92.11%), and with ≤ 1:4 maternal titers (84.59%). In 2020, the proportions of maternal syphilis among women aged below 20 years(4.52%) or ≥ 35 years(22.23%), multipara (60.55%), women with pregnancy complications (19.60%) highly increased. However, the proportion of maternal syphilis among women with stable marital status (89.98%) decreased significantly (Table 2).

**Table 2:** Changes in maternal characteristics with syphilis

Variable		2015	2016	2017	2018	2019	2020	total	χ^2^	*P*
Maternal age										
< 20 years old	N	82	72	69	50	60	93	426	80.186	** < 0.001**
	%	3.68	2.86	2.61	2.23	2.81	4.52	3.08		
≥ 35 years old	N	343	417	552	438	448	457	2655		
	%	15.37	16.55	20.85	19.55	20.98	22.23	19.20		
Unemployed	N	1134	1288	1372	1107	1115	1043	7059	4.342	0.501
	%	50.83	51.11	51.83	49.42	52.22	50.73	51.04		
Education										
Primary	N	333	346	324	267	325	276	1871	43.626	** < 0.001**
	%	14.93	13.73	12.24	11.92	15.22	13.42	13.53		
College	N	183	259	265	246	239	263	1455		
	%	8.20	10.28	10.01	10.98	11.19	12.79	10.52		
Middle	N	1715	1915	2058	1727	1571	1517	10,503		
	%	76.87	75.99	77.75	77.10	73.58	73.78	75.95		
Multipara	N	1170	1438	1587	1322	1285	1245	6802	43.586*	** < 0.001**
	%	52.44	57.06	59.95	59.02	60.19	60.55	57.78		
Marital status	N	2047	2321	2474	2089	1957	1850	12,738	24.560	** < 0.001**
	%	91.75	92.10	93.46	93.26	91.66	89.98	92.11		
Local residents	N	1222	1508	1503	1241	1053	998	7525	87.867*	** < 0.001**
	%	54.77	59.84	56.78	55.40	49.32	48.54	54.41		
Pregnancy complications	N	367	407	441	368	372	403	2358	12.262*	**0.031**
	%	16.45	16.15	16.66	16.43	17.42	19.60	17.05		
Maternal RPR/TRUST Serological titer (≤ 1:4)	N	1803	2129	2225	1926	1852	1763	11,698	37.024*	** < 0.001**
	%	80.82	84.48	84.06	85.98	86.74	85.75	84.59		

^*^P for trend

### Changes in syphilis treatment

During 2015–2020, we observed a dramatic change in the overall treatment coverage, increasing from 88.30% to 98.25% (APC = 2.37, 95%CI: 1.63 ~ 3.12, P = 0.001). Also, the coverage of adequate treatment gradually increased from 66.92% to 83.37% (APC = 4.84, 95%CI: 3.49 ~ 6.21, P = 0.001).It is worth mentioning that the gap between locals and migrants was narrowed (all P < 0.001). (Table3).

**Table 3:** Changes in maternal syphilis treatment between women living in Zhejiang or coming from other provinces

Year	Number of women with syphilis	Overall treatment		Adequate treatment		Zhejiang	Overall treatment		Adequate treatment		Other provinces	Overall treatment		Adequate treatment	
		Number	Rate (%)	Number	Rate (%)		Number	Rate (%)	Number	Rate (%)		Number	Rate (%)	Number	Rate (%)
2015	2231	1970	88.30	1493	66.92	1222	1107	90.59	892	73.00	1009	863	85.53	601	59.56
2016	2520	2261	89.72	1715	68.06	1509	1366	90.52	1084	71.84	1011	895	88.53	631	62.41
2017	2647	2467	93.20	1932	72.99	1503	1408	93.68	1124	74.78	1144	1059	92.57	808	70.63
2018	2240	2154	96.16	1762	78.66	1241	1194	96.21	995	80.18	999	960	96.10	767	76.78
2019	2135	2084	97.61	1702	79.72	1053	1026	97.44	864	82.05	1082	1058	97.78	838	77.45
2020	2056	2020	98.25	1714	83.37	997	984	98.70	837	83.95	1059	1036	97.83	877	82.81
APC (95%CI)		2.37(1.63, 3.12)		4.84(3.49, 6.21)			1.99(1.33, 2.65)		3.46(1.87, 5.08)			2.88(1.73, 4.04)		6.89(4.63, 9.20)	
P		< 0.001		< 0.001			< 0.001		< 0.001			< 0.001		< 0.001	

### Changes in pregnancy outcomes and risk factors

Over the years, no significant decrease was observed in the overall incidence of APOs (APC = 0.16, 95%CI: -2.57 ~ 2.97, P = 0.877); however,the incidences of perinatal death(APC = -19.30, 95%CI: -24.33 ~ -13.92, P = 0.001),CS (APC = -26.55, 95%CI: -38.75 ~ -11.92, P = 0.009), and other neonatal complications; birth defect, neonatal pneumonia, asphyxia neonatorum (APC = -14.67, 95%CI: -23.96 ~ -4.24, P = 0.019) decreased significantly. In 2020, the incidences of perinatal death, CS and other APOs fell to the lowest level, 6.14‰, 2.36‰, and 0.57%, respectively (Table 4).

**Table 4:** Changes of APOs incidences by categories during 2015–2020

Year	Number of birth	Overall APOs		LBW/preterm birth		Perinatal death		Congenital syphilis		Other neonatal complications	
		n	%	n	%	n	‰	n	‰	n	%
2015	2246	259	11.53	251	11.18	42	18.70	18	8.01	28	1.25
2016	2539	288	11.34	261	10.28	36	14.18	23	9.06	37	1.46
2017	2689	282	10.49	251	9.33	29	10.78	15	5.58	33	1.23
2018	2273	258	11.35	243	10.69	25	11.00	7	3.08	21	0.92
2019	2196	246	11.20	241	10.97	16	7.29	5	2.28	18	0.82
2020	2116	245	11.58	205	9.69	13	6.14	5	2.36	12	0.57
APC(95% CI)		0.16(-2.57, 2.97)		-1.00(-6.02, 4.28)		-19.30(-24.33, -13.92)		-26.55(-38.75, -11.92)		-14.67(-23.96, -4.24)	
P		0.877		0.618		**0.001**		**0.009**		**0.019**	

**Fig. 1:**
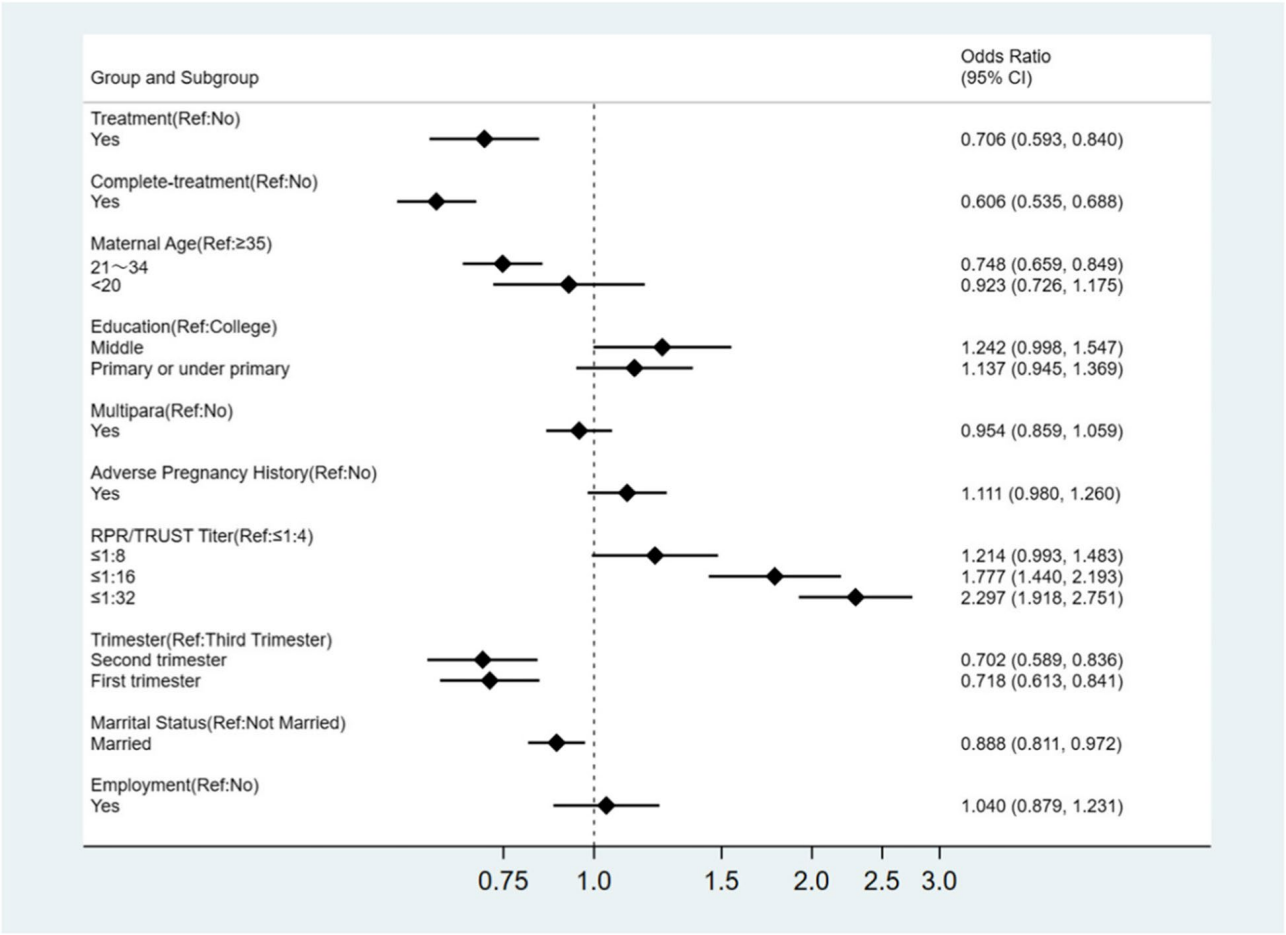
Predictors for adverse pregnancy outcomes by multiple logistic mode

Maternal syphilis treatment and adequacy treatment were protective factors for APOs (OR_adj_ = 0.71 95%CI: 0.59 ~ 0.84, P < 0.001; OR_adj_ = 0.61 95%CI: 0.54 ~ 0.69, P < 0.001). The APOs risk was significantly decreased among women aged between 21 and 34 years compared to those aged 35 years and above (OR_adj_ = 0.75 95%CI: 0.66 ~ 0.85, P < 0.001). It is worth noting that risk of APOs increased with increasing maternal RPR/TRUST titers. Compared to women with titers ≤ 1:4, women with titers ≥ 1:32 (OR:2.30,95%CI 1.92 ~ 2.75) and those with titers 1:16(OR:1.78,95% CI 1.44 ~ 2.19) had significantly increased risk of APOs. However, women who received first ANC in first trimester (OR: 0.72, 95%CI 0.61 ~ 0.84) and second trimester (OR: 0.70,95% CI 0.59 ~ 0.84) had significantly reduced risk of APOs compared to women who had first ANC in the third trimester. Additionally, stable marriage was a protective factor against the risk of APOs(ORadj = 0.89 95%CI: 0.81 ~ 0.97) (Table 5, Fig. 1).

**Table 5:** Predictors for adverse pregnancy outcomes by multiple logistic mode

Variable	B	Wald	P	OR_adj,_95% CI
Treatment	-0.35	15.33	** < 0.001**	0.71(0.59 ~ 0.84)
Complete-treatment	-0.50	60.98	** < 0.001**	0.61(0.54 ~ 0.69)
Maternal age				
≥ 35 years old		22.26	** < 0.001**	
< 20 years old	-0.08	0.42	0.516	0.92(0.73 ~ 1.18)
21 ~ 34 years old	-0.29	20.06	** < 0.001**	0.75(0.66 ~ 0.85)
Education				
College		3.81	0.149	
Middle	0.22	3.76	0.052	1.24(1.00 ~ 1.55)
Primary or under primary	0.13	1.84	0.175	1.14(0.95 ~ 1.37)
Multipara	-0.05	0.777	0.378	0.95(0.86 ~ 1.06)
Adverse pregnancy history	0.11	2.69	0.101	1.11(0.98 ~ 1.26)
RPR/TRUST				
≤ 1:4		101.33	** < 0.001**	
≥ 1:32	0.83	81.68	** < 0.001**	2.30(1.92 ~ 2.75)
1:16	0.58	28.77	** < 0.001**	1.78(1.44 ~ 2.19)
1:8	0.19	3.58	0.058	1.21(0.99 ~ 1.48)
First ANC				
Third trimester		18.91	** < 0.001**	
First trimester	-0.33	16.79	** < 0.001**	0.72(0.61 ~ 0.84)
Second trimester	-0.35	15.70	** < 0.001**	0.70(0.59 ~ 0.84)
Married	-0.12	6.58	**0.010**	0.89(0.81 ~ 0.97)
Employment	0.04	0.21	0.648	1.04(0.88 ~ 1.23)

## Discussion

This study indicates improvement in syphilis screening and treatment in pregnant women in Zhejiang province, especially the narrowed gap between local residents and migrants. Interestingly, it shows decreased incidence of APOs and improved newborn outcomes over the years. In 2020, nearly all pregnant women received syphilis screening, 98.25% received treatment, and CS incidence dropped to a low level, meeting the global goals of EMTCT [4, 6, 7].

Globally, coverage of syphilis testing increased from 59 to 66%, and maternal syphilis treatment increased from 74 to 78% between 2012 and 2016 [1]. At Chinese national level, treatment coverage reached 89.52% in 2019 [10]. Remarkable achievements in EMTCT of syphilis have also been reported in most areas in China and worldwide [1, 14–16]. Findings in our study reflected persistent efforts on EMTCT as a pilot areas in China, particularly contributing to strong policy support, strengthened package of ANC and EMTCT services, sustainable provision of penicillin and quality of data collection.

In Zhejiang province, EMTCT has been integrated with ANC services for pregnant women and funded by government. Rapid syphilis screening is available in primary care facilities. Hospitals qualified to test and treat maternal syphilis are over all counties. Therefore, during 2015 to 2020, coverage of ANC in the first trimester increased by 6.71% annually in our study, which promoted increase in coverage of treatment as well. In regard to migrants, we noted 6.89% per year increase in full treatment.

It is important to note the challenges of ANC and treatment lack or delay among women with syphilis worldwide [1]. Since 2017, we gave case reviews on women who had delayed early ANC, inadequate treatment or vertical transmission. poor awareness of ANC in pregnant women, limited EMTCT knowledge in healthcare providers, vulnerabilities in laboratory facilities as reported elsewhere were barrier for EMTCT [17–23].

Maternal syphilis incidence remained high in Zhejiang. reaching 0.44% in 2020. Our estimate was far higher than the average level in Netherlands (0.06%–0.08%) and Beijing(China) (1.4‰) in 2015, however much lower than the data in African regions (1.52%), Region of the Americas(0.86%), Eastern Mediterranean Region(0.77%) in 2016 [1, 18, 22]. The epidemic of maternal syphilis in this study was also slightly expanded compared to the previous study of Zhejiang in 2013–2014 [13]. As the global maternal syphilis remained stable in most area, the growth of syphilis in pregnant women in Zhejiang should be given serious consideration [1, 10, 13]. The increasing syphilis screening in pregnant women might partly explained the rising incidence. Moreover, it was also a specific example of increasing long term trend in syphilis in China [15]. One step of screening and treatment package widely suggested should be considered [17, 24].

We evaluated the characteristics of women with syphilis in order to develop target interventions. The proportion of women with advanced age(≥ 35 years) increased greatly, exceeding 20%.The rapid development of social-economy, work and lifestyle pressure,might force women to put off giving birth, particularly since the new birth policy changes in China [25, 26]. Similarly, reports indicated that women with syphilis infection were more likely to be illiterate, migrants and multiparous at regional and national level [10, 21, 22, 27]. Furthermore, a rise in pregnancy complications proportion in women with syphilis might be the consequence of increasing number of women with advanced age and higher parity. This point highlighted the need for the potential improved risk management for APOs.

The average incidence of APOs at our national level was 13.82% in 2016–2019, reflecting fetal loss or stillbirth, as well as abnormal infant parameters. In Guangzhou, this figure was 27.3% during 2011–2018, including ectopic pregnancy, spontaneous abortion, stillbirth, prematurity or LBW, a live infant birth weight of less than the 10^th^ percentile by gestational age and sex, infant death and CS [28]. Therefore, differences in inclusion and exclusion criteria in different studies while comparing the overall occurrences of APOs should be given consideration. Exclusion of early fetal loss in our study resulted in decreased incidence of APOs for this reason the incidence of APOs might be underestimated. LBW/preterm birth was the most common subtypes of APOs, which was shown in previous studies [4, 10, 28]. No significant decline in LBW/preterm birth was possible for the composite negative effect from increasing maternal age and pregnancy complications. The obvious reduction of APOs categories were predominantly in CS, abnormal signs and perinatal death. Our hypothesis indicates increasing coverage and effectiveness of testing, early ANC, and treatment. Women with treatment, especially adequate treatment, early ANC and appropriate maternal age were less likely to experience APOs. APOs risk increased with maternal RPR/TRUST titers, which was significantly severe among women with atiter of ≥ 1:32 at delivery or third trimester. The above evidences have been widely reported, and persisting low nontreponemal titers (< 1:8) is frequently suggested [10, 18, 28].

This study has several limitations. Firstly, we focused on selected APOs. Missing information on early fetal loss and abortion could lead to some selection bias to the comprehensive understanding of APOs associated with syphilis. Secondly, some risk factors, such as the mother's stage of syphilis infection, gestational age at syphilis status, mode of delivery, sexual partner’s infection status and congenital birth defects, were not considered [5, 10, 29]. Lastly, Zhejiang is a province with rapid economic growth and with qualified healthcare system. The lessons from Zhejiang's experiences need to be considered cautiously by other less developed regions.

## Conclusions

This study included a substantial number of pregnant women, thus providing a powerful and effective detection of maternal syphilis and associated APOs. In summary, expanding the coverage of syphilis screening, early ANC, and adequate treatment will be the most effective way to improve pregnancy outcomes and promote the health condition of infants born to maternal syphilis, even in a high endemic region.

## Data Availability

Data are available upon reasonable request. The Study group will review proposals. Please contact Corresponding author, Dr Xiaohui Zhang (zjfb_amy@zju.edu.cn).
